# Stretch stress propels glutamine dependency and glycolysis in optic nerve head astrocytes

**DOI:** 10.3389/fnins.2022.957034

**Published:** 2022-08-05

**Authors:** Nathaniel Pappenhagen, Eric Yin, Autumn B. Morgan, Charles C. Kiehlbauch, Denise M. Inman

**Affiliations:** ^1^Department of Pharmaceutical Sciences, North Texas Eye Research Institute, University of North Texas Health Science Center, Fort Worth, TX, United States; ^2^Texas College of Osteopathic Medicine, University of North Texas Health Science Center, Fort Worth, TX, United States

**Keywords:** astrocyte, glaucoma, optic nerve head, metabolism, glycolysis

## Abstract

Glaucoma is an optic neuropathy that leads to irreversible blindness, the most common subtype of which is typified by a chronic increase in intraocular pressure that promotes a stretch injury to the optic nerve head. In rodents, the predominant glial cell in this region is the optic nerve head astrocyte that provides axons with metabolic support, likely by releasing lactate produced through astrocytic glycolysis. Our primary hypothesis is that stretching of the optic nerve head astrocytes alters their metabolic activity, thereby advancing glaucoma-associated degeneration by compromising the metabolic support that the astrocytes provide to the axons in the optic nerve head. Metabolic changes in optic nerve head astrocytes were investigated by subjecting them to 24 h of 12% biaxial stretch at 1 Hz then measuring the cells’ bioenergetics using a Seahorse XFe24 Analyzer. We observed significant glycolytic and respiratory activity differences between control and stretched cells, including greater extracellular acidification and lower ATP-linked respiration, yet higher maximal respiration and spare capacity in stretched optic nerve head astrocytes. We also determined that both control and stretched optic nerve head astrocytes displayed a dependency for glutamine over pyruvate or long-chain fatty acids for fuel. The increased use of glycolysis as indicated by the extracellular acidification rate, concomitant with a dependency on glutamine, suggests the need to replenish NAD + for continued glycolysis and provision of carbon for TCA cycle intermediates. Stretch alters optic nerve astrocyte bioenergetics to support an increased demand for internal and external energy.

## Introduction

Glaucoma is an optic neuropathy that is the largest cause of irreversible blindness in the world (Weinreb et al., 2014). The optic nerve head (ONH) is the initial site of degeneration in glaucoma as demonstrated in human glaucomic patients (Quigley et al., 1981), as well as rodent glaucoma models (Howell et al., 2007; Chidlow et al., 2011). Loss of axon transport and axonal loss at the ONH is present in many *in vivo* models of glaucoma (Guo et al., 2005; Chidlow et al., 2011; Wilson et al., 2016; Maddineni et al., 2020), indicating that the ONH is an important region for understanding glaucoma. The distension of the eye through the lamina cribrosa causes the optic disk to press back through the lamina cribrosa of the ONH, leading to an estimated chronic 9% stretch (Girard et al., 2016). This optic cupping accompanies RGC loss, axonal transport loss, and visual field loss. Optic nerve head astrocytes (ONHAs) react to this stretch in glaucoma by entering a state of reactivity and hypertrophy (Hernandez, 2000a; Sun et al., 2013) that impacts the health of the retinal ganglion cells (RGCs) and their axons (Schneider and Fuchshofer, 2015). Reactive astrocytes negatively impact neurons through the generation of pro-inflammatory molecules (Neufeld and Liu, 2003; Oikawa et al., 2020), deposition of extracellular matrix (Morgan, 2000), and changes in metabolic support that can impact axonal transport, mismanage metabolism, and lead to degeneration (Chung et al., 2009; Vohra et al., 2013; Iglesias et al., 2017).

We have demonstrated changes in metabolic transporter profiles in retina and optic nerve from glaucoma subjects (Harun-or-Rashid et al., 2018). Glucose transporter 1 (GLUT1), the primary glucose transporter found on epithelial cells and astrocytes (Morgello et al., 1995), is decreased in the optic nerve (ON) in a chronic mouse model of glaucoma (Harun-or-Rashid et al., 2018). Additionally, monocarboxylate transporters that move lactate, pyruvate, and ketone bodies (Halestrap, 2012) across membranes, are decreased in glaucomic ON. Downregulation of transporter proteins suggest metabolic shifts accompany glaucomic degeneration because glial cells and RGCs form a metabolic unit of cells that operate as a mutually supportive entity to meet energy needs (Tsacopoulos and Magistretti, 1996). The lactate shuttle hypothesis proposes that glial glycolysis produces lactate to be exported to neighboring neurons for conversion to pyruvate and as fuel for mitochondrial respiration (Pellerin et al., 1998; Volkenhoff et al., 2015; Saab et al., 2016; Muraleedharan et al., 2020). Glucose entry to this metabolic unit would be decreased by the loss of GLUT1, and loss of MCT-1, -2, or -4 would ultimately limit the energy availability in the RGC axons in the ON, which is detrimental for the health of the glaucomic nerve.

Altering metabolic activity in mouse models of glaucoma has had protective effects for RGCs, suggesting that provision of adequate sources of energy can sustain RGC survival (Harun-Or-Rashid et al., 2020b; Tribble et al., 2021). We have increased metabolic activity in glaucomic animals by using a ketogenic diet to increase mitochondrial activity and by using an adeno-associated virus to increase MCT-2 expression, thereby increasing metabolic substrate availability in glaucomic tissues (Harun-or-Rashid et al., 2018; Harun-Or-Rashid et al., 2020a). Additionally, restoration of nicotinamide adenine dinucleotide (NAD^+^), a critical electron carrying molecule for the citric acid cycle and glycolysis, to glaucomatous retina with administration of vitamin B_3_ protects RGCs (Williams et al., 2017). Providing pyruvate or nicotinamide directly in the drinking water preserved RGC number and positively impacted inner retina function in the DBA/2J model of glaucoma (Harder et al., 2020). Promisingly, treatment of glaucoma patients with a nicotinamide supplement increased inner retina function (Hui et al., 2020). Our lab has shown that glaucomic ON has a higher glycolytic rate and impaired glycolytic responsiveness than control ON by measuring the extracellular acidification rate (ECAR) as well as measuring glycolytic increase after inhibiting mitochondrial ATP production in *ex vivo* ON (Jassim et al., 2019). In this study, we move from the ON to the ONH to evaluate the metabolic changes in ONHAs that have been subjected to a glaucoma-associated stretch injury. Previous investigation of basic ONH metabolism has been limited to human lamina cribrosa cells, which were observed to increase glycolysis concomitant with reduced oxidative phosphorylation in glaucoma patients (Kamel et al., 2020). Our findings in ONH astrocytes show greater capacity for oxidative phosphorylation when cells are placed under stretch. We also observe increases in glycolysis in stretched cells, and find that ONH astrocytes have a preference for glutamine to meet their energy needs.

## Materials and methods

### Primary optic nerve head astrocyte isolation

Primary optic nerve head astrocytes (ONHAs) were isolated from postnatal day (P) 5-7 rat pups (CD (Sprague Dawley) IGS rats from Charles River Laboratories, Wilmington, MA, United States) using modified methods (Kaja et al., 2015). P5-7 rat pups were sacrificed by decapitation and their eyes and ON were harvested. All procedures were approved by the Institutional Animal Care and Use Committee and performed in accordance with the ARVO Statement for the Use of Animals in Ophthalmic and Vision Research. Individual optic nerve head explants were placed into a 24-well plate, and allowed to adhere before 1 mL of astrocyte growth media (DMEM with 4.5 mg/mL glucose (Gibco through Fisher Scientific, Cat. No. 11995073), 2 mM glutamine, 1 mM sodium pyruvate, 1x penicillin/streptomycin and 10% heat inactivated-fetal bovine serum, [ThermoFisher Scientific, Cat. No. 16140063)] was added to the explants. Astrocytes grew out from the explants for 5–7 days before they were passaged using TrypLE (Gibco through Fisher Scientific, Cat. No. 50-591-419), with additional filtration through a sterile 100 μm filter before centrifugation, TrypLE removal, then plating in T75 flasks. In the flasks, primary astrocytes were grown to 75% confluence then shaken at 200 RPM for 30 min at room temperature to remove any non-astrocytic cells. ONH astrocytes (150,000 per well) were then seeded on 6-well FlexCell culture plates coated with 200 μg/mL type 1 collagen for the stretch experiments (FlexCell International, Cat. No. BF-3001C).

### Stretch experiments

Primary cells grown to 75% confluency were stretched in a manner similar to that experienced in the optic nerve head (ONH) in glaucoma (Downs et al., 2008; Girard et al., 2016) using the FlexCell 6000T which uses vacuum to pull the membrane on which the cells are seeded over a plastic post, thus stretching them biaxially. We used a 12% sinusoidal stretch protocol with a 1 Hz frequency for 24 h to imitate glaucomic stretch in the cultured ONH astrocytes, and emulate other published research done on mechanical stretch in primary ONH cells and astrocytes (Rogers et al., 2012a,b; Albalawi et al., 2017). Control primary cells were grown on 6-well FlexCell plates coated with 200 μg/mL type 1 collagen until 75% confluency before being collected for the Seahorse experiments.

### Seahorse XFe 24 analyzer experiments

The Seahorse XFe24 Analyzer (Agilent Technologies, Santa Clara, CA, United States) measures oxygen and pH in mitochondria, cells or tissue using fiber optic sensors. Oxygen consumption rate allows the user to estimate mitochondrial respiration while released protons can be used to estimate lactate release, a measure of glycolytic activity. Injection ports for each culture well enable challenges of cellular metabolic processes through the addition of inhibitors of electron transport chain complexes. We used the glycolytic rate assay, the mitochondrial fuel flex test, and the mitochondrial stress test protocols to measure the metabolic changes in ONHAs following glaucoma-like stretch. Seeding optimization for the initial Seahorse experiments proceeded by seeding at densities from 10,000 to 50,000 cells per well, then evaluating baseline oxygen consumption rate. The most reliable oxygen consumption rate was obtained with 50,000 cells per well, so that is the cell density used throughout these experiments. Data points in all graphs represent data from individual wells in the Seahorse plate. The experiments were repeated 5-6 times using biological replicates (separate ONH astrocyte isolations from different rat litters).

CellTak-coated Seahorse plates were prepared prior to the addition of the ONH astrocytes by incubating the plates with 22.4 μg/mL CellTak (Corning, through Fisher Scientific, Cat. No. 354240) for 20 min before rinsing twice with sterile ddH_2_O. Once the FlexCell stretch protocol was complete, ONH astrocytes were removed from the membranes with TrypLE, resuspended in 1 mL of the appropriate Seahorse assay media (DMEM with 2 mM glutamine for the glycolytic stress test, or DMEM with 2 mM Glutamine, 1 mM sodium pyruvate, and 1 mM glucose for the mitochondrial stress test, mitochondrial fuel dependency test, or mitochondrial fuel flexibility test), and seeded at 50,000 cells per well on the CellTak-coated Seahorse culture plates. The plates were spun at 200 g for 1 min with no braking then incubated without CO_2_ at 37°C for 25 min before the final 400 μL of Seahorse assay media was added to each well in preparation for the Seahorse experiment.

### Glycolytic stress test

For the modified glycolytic stress test, cells were assayed in DMEM media with only 2 mM glutamine, depriving the cells of glucose to allow for a baseline measurement of the extracellular acidification rate (ECAR). Once glucose is added, the difference between baseline and post-glucose injection is the ECAR associated with glycolysis. All inhibitor compounds are from Sigma-Aldrich, St. Louis, MO, United States, unless otherwise specified. With the injection of 0.5 μM rotenone (Cat. No. R8875) and 0.5 μM antimycin-a (Cat. No. A8674), Complex I and Complex III inhibitors, respectively, mitochondrial respiration in the cells ceases while glycolytic activity and ECAR increase due to metabolic shift from the loss of mitochondrial adenosine triphosphate (ATP) production. Finally, addition of excess 2-deoxyglucose (2-DG, 50 mM, Cat. No. D8375), a non-hydrolyzable form of glucose, competitively inhibits hexokinase, thereby stopping glycolysis and restoring cells to baseline ECAR. The maximal glycolytic rate is defined as the maximal ECAR minus the baseline ECAR, and the glycolytic reserve is the maximal ECAR minus the glycolytic ECAR.

Cells were removed following the glycolytic rate assay, fixed for 15 min at 37°C with 2% formaldehyde, labeled with 4′,6-diamidino-2-phenylindole (DAPI, Cat. No. D9542) in Tris-buffered saline (TBS) for 10 min, then rinsed with PBS with 0.1% sodium azide until the cells were counted for assay data normalization.

### Mitochondrial stress test

The mitochondrial stress test measures oxygen consumption rate (OCR) in response to serial injection of an inhibitor of ATP synthase (oligomycin-a, Cat. No. 75351), a protonophore (trifluoromethoxy carbonylcyanide phenylhydrazone, FCCP, Cat. No. C2920), and electron transport chain Complex I and III inhibitors (rotenone and antimycin-a). The media used was DMEM with 1 mM glucose, 1 mM sodium pyruvate, and 2 mM glutamine. After baseline OCR measures, an injection of oligomycin (1 mM) typically halts mitochondrial respiration-associated ATP production and the OCR falls; the difference between baseline OCR and OCR during oligomycin-a exposure is considered the oxygen consumption attributable to ATP production. The protonophore FCCP (2 mM), by uncoupling the mitochondrial membrane potential from ATP production, leads to an increase in oxygen consumption (maximal respiration) as the electron transport chain (ETC) tries to maintain the proton gradient. Finally, rotenone and antimycin-a (0.5 μM each) inhibit Complexes I and III, respectively, thereby completely shutting down respiration-associated OCR. Following the mitochondrial stress test, cells were fixed and labeled with DAPI as previously described.

### Mitochondrial fuel dependency

The mitochondrial fuel dependency test was used to test the reliance of the mitochondria on three of the most common mitochondrial substrates; pyruvate, glutamine, and long chain fatty acids. The assay was run in triplicate on a single plate, with 6-7 wells dedicated to each fuel dependency. All compounds for the dependency tests were obtained from Sigma-Aldrich (St. Louis, MO, United States). The assay used the mitochondrial pyruvate carrier inhibitor 2-cyano-3-(1-phenyl-1H-indol-3-yl)-2-propenoic acid (UK5099, Cat. No. PZ0160); an allosteric inhibitor of glutaminase, bis-2-(5-phenylacetamido-1,3,4-thiadiazol-2-yl)ethyl sulfide (BPTES, Cat. No. SML0601); and the carnitine palmitoyl-transferase 1A inhibitor etomoxir (Cat. No. 236020) to inhibit long chain fatty acid transport into the mitochondria. To determine the mitochondrial dependency on each fuel source, we followed instructions as outlined in the Agilent Seahorse XF Mito Fuel Flex Test Kit. To summarize, changes in OCR are measured after addition of a pathway inhibitor followed by the addition of the other inhibitors. For example, to measure pyruvate dependence, baseline OCR is measured then UK5099 is added; OCR change is recorded, then BPTES and etomoxir (inhibitors of the glutamine and long chain fatty acid pathways, respectively) are added. The difference in the UK5099 OCR from baseline divided by and the other inhibitors’ OCR compared to baseline yields the dependency measure; the equation used: Dependency% = [Baseline OCR – Target Inhibitor OCR/Baseline OCR – All Inhibitors OCR] *100.

### Protein isolation and analysis

Optic nerve head astrocyte (ONHA) protein was isolated from control and stretched primary cells after removal from FlexCell plates using TrypLE followed by lysis in tissue protein extraction reagent (T-PER, ThermoFisher, Waltham, MA, United States, Cat. No. PI78510) containing HALT protease and phosphatase inhibitors (ThermoFisher, Cat. No. 78442). Protein lysates collected from four separate experiments were subjected to three pulses of sonication (10% amplitude), with a few minutes rest between pulses, then centrifugation at 10,000 *g* for 10 min. Supernatants were collected and protein concentration determined through BCA assay (Pierce through ThermoFisher, Cat. No. PI23225). Proteins were quantified using the Jess capillary electrophoresis instrument from ProteinSimple (San Jose, CA, United States), and normalized to total protein. For each ONH astrocyte sample, a “no primary antibody” negative control was included, as well as a positive control consisting of mouse retinal protein. Protein-antibody interactions were optimized by varying the protein and antibody concentrations until clear, strong, specific binding was observed as denoted by a single peak at the correct molecular weight for the protein in question. All antibodies were tested at 1:25, 1:50, and 1:100 concentrations across protein concentrations of 0.2, 0.4, 0.8, and 1.2 μg/μL. Chemiluminescence plots with the highest peaks were identified and the antibody-protein concentrations from those combinations were used. Antibodies used were LDH-A (Novus Biologicals, Centennial, CO, United States, NBP1-48336, RRID:AB_10011099), GFAP (Abcam, Cambridge, MA, United States, Cat# ab53554, RRID:AB_880202), GFAP (Millipore, Burlington, MA, United States, Cat# MAB3402, RRID:AB_94844), GLUT-1 (Novus Biologicals, Centennial, CO, United States, NB110-39113, RRID:AB_1851003), Glutamine synthetase (Santa Cruz Biotechnology, Dallas, TX, United States, sc-74430, RRID:AB_1127501), GLAST (Novus Biologicals, NB100-1869, RRID:AB_531518), S100β (Sigma-Aldrich Cat# S2644, RRID:AB_477501), glucose-6-phosphate dehydrogenase (Cell Signaling Technology, Danvers, MA, United States, 8866, RRID:AB_10827744), Iba1 (FUJIFILM Wako Shibayagi, Richmond, VA, United States, Cat# 019-19741, RRID:AB_839504), and β-tubulin (Covance Cat# PRB-435P-100, RRID:AB_291637).

### mRNA isolation and analysis

All reagents for mRNA isolation and analysis were obtained from ThermoFisher Scientific (Waltham, MA, United States) unless otherwise specified. ONH astrocytes were grown to confluence, media was removed and Trizol (Invitrogen through ThermoFisher Scientific, Cat. No. 15596026) added to the flask. Cells in Trizol were scraped together and collected into a 2 mL tube. Isolation of mRNA proceeded according to the manufacturer’s instructions for Trizol. Briefly, the cells were pipetted up and down to shear, then rested for 5 min for nucleoprotein complex dissociation before addition of chloroform. After 3 min incubation, samples were centrifuged for 15 min at 14,000× *g* at 4°C. The resultant upper aqueous phase was transferred to a new tube and isopropanol was added to precipitate the RNA. The samples were incubated for 10 min then centrifuged for 10 min at 14,000× *g* at 4°C. The pellet was washed 2× in 75% ethanol then resuspended in 20 uL RNase-free water (Cat. No. AM9937). Isolated mRNA was converted to cDNA using the Verso cDNA Synthesis Kit (Cat. No. AB1453B). For qPCR, cDNA was diluted to 5 ng/uL, combined with TaqMan Universal PCR Master Mix (Cat. No. 4440040) and one of the following TaqMan assays (Cat. No. 4331182, see below) then loaded onto a QuantStudio-5 384-well real-time PCR system (Applied Biosystems through ThermoFisher Scientific, Cat. No. A28575). Samples were run in triplicate. The housekeeping gene was β-actin. The endogenous control was cDNA from a 2 month-old mouse retina. Fully validated TaqMan assays used in this analysis: *Actb* (Mm02619580_g1), *Gfap* (Mm01253033_m1), *Glud1* (Mm00492353_m1), *Glul* (Mm00725701_s1), *Slc1a3* (Mm00600697_m1), *Rbpms* (Mm00803908_m1), *Mbp* (Mm01262037_m1), *Iba1* (Mm00479862_g1), *Gja1* (Mm00439105_m1).

### Immunolabeling of cells and tissue

Optic nerve head (ONH) astrocytes grown on coverslips were fixed in pre-warmed 3% paraformaldehyde in minimal media for 15 min at 37°C. Coverslips were rinsed 2× in 0.1M phosphate buffered saline (PBS) before aldehydes were quenched during 30 min incubation with 50mM ammonium chloride. Cells were permeabilized for 10 min with 0.1% Triton X-100 in 0.1M PBS, then rinsed 2× in 0.1M PBS. Cells were blocked in 5% bovine serum albumin + 5% donkey serum in 0.1M PBS for 2 h at room temperature. Block was removed and primary antibodies added for 2 h at RT. Primary antibody information is listed above in the section on Protein Isolation and Analysis. Cells were rinsed 1× in block solution prior to addition of secondary antibodies for 1h at RT. Secondary antibodies were species-specific secondary antibodies conjugated to AlexaFluor-488 or -594, or -647 (Jackson ImmunoResearch, Cat. No.s 711-545-152, 703-585-155, and 715-605-151). Cells were rinsed 3x in 0.1M PBS then coverslipped with DAPI-Fluoromount-G (SouthernBiotech, Cat. No. 0100-20).

Eye globes with attached optic nerves were obtained from a 2 month-old mouse that had been trans-caridally perfused with 4% paraformaldehyde. After cryoprotection in 30% sucrose for 24 h, the lens and cornea were removed from the globes and then they were frozen using isopentane-cooled liquid nitrogen in Tissue-Tek OCT Compound (Sakura Finetek, Torrance, CA, United States, Cat. No. 4583). Globes were sectioned at 20 μm using a Leica CM-1510 cryostat and collected on SuperFrost charged glass slides (Fisher Scientific, Cat. No. 12-550-15). Immunolabeling proceeded after frozen tissue was thawed for 20 min at RT, followed by 3 × 10 min rinses in 0.1M PBS. Slides were blocked for 1 h in 5% donkey serum and 0.4% Triton X-100 in 0.1M PBS then incubated in primary antibodies (see above) overnight at 4°C. After washing 3× for 10 min each with 0.1M PBS, slides were incubated in block for 30 min then incubated for 2 h in secondary antibodies (described above). A final series of 0.1M PBS washes were followed by coverslipping the slides in DAPI-Fluoromount-G.

Cells and tissue were imaged using a Leica DMi8 confocal microscope. Laser intensity and exposure settings were kept consistent across groups within cell or tissue imaging sessions.

### Data normalization

Oxygen consumption rate (OCR) and ECAR values were normalized to DAPI-labeled cell number in each well as quantified using the Cytation 5 (Biotek) cell imaging multi-mode reader and its automated counting feature. Five images at 4× were taken per well, and images were examined to exclude aberrations such as cell clusters in which individual nuclei could not be resolved. Average cell density of each image was used to calculate the number of cells present in each well. Normalized OCR and ECAR data were analyzed in GraphPad Prism v. 9.

### Statistical analysis

Unpaired two-tailed student’s *t*-tests were used to compare control and stretched ONH astrocytes, with Mann-Whitney rank comparisons when necessary for data that was not normally distributed. Fuel dependency data (Figure 4) was analyzed by one-way ANOVA with Tukey’s multiple comparisons test to compare individual groups. Comparisons for which *p* < 0.05 were considered statistically significant. Statistical analysis was done using GraphPad Prism 9. Data points in all bioenergetic analysis graphs represent data from individual wells in the Seahorse plate. Experiments were repeated 5-6 times using biological replicates (separate ONH astrocyte isolations from different rat litters).

**FIGURE 1:**
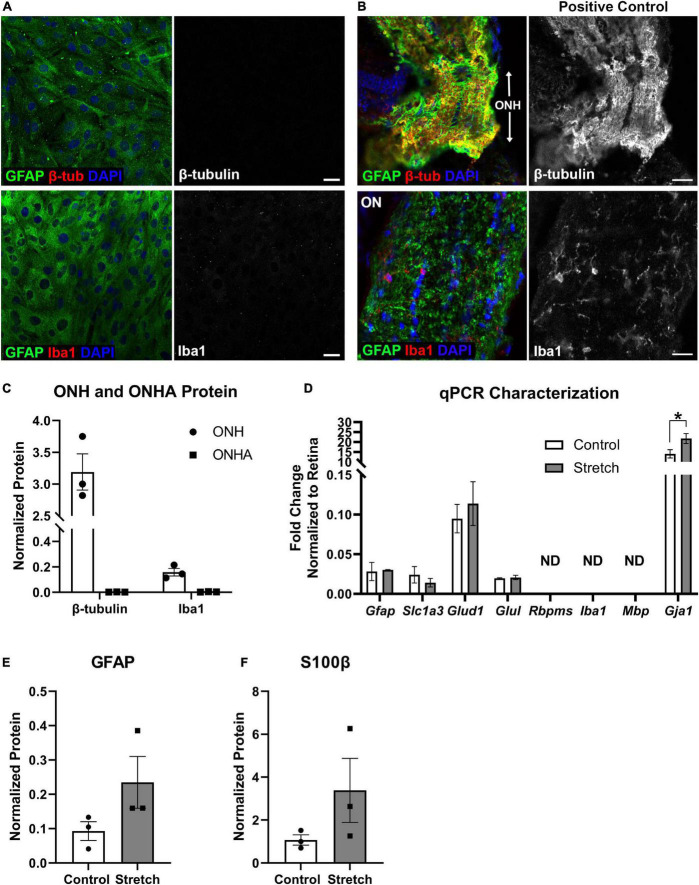
*Optic nerve head astrocyte characterization*. **(A)** Isolated optic nerve head (ONH) astrocytes were immunolabeled using antibodies against GFAP (green), β-tubulin (red, upper panels), and Iba1 (red, lower panels) to determine the purity of the cell cultures. DAPI was used to stain cell nuclei (blue). Neither β-tubulin nor Iba1 was detected in the cultures. Scale bar = 10 μm. **(B)** Sections of retina with ONH and optic nerve attached served as positive controls for the immunolabeling in **(A)**. Both β-tubulin and Iba1 were observed in the mouse ONH and optic nerve. Scale bar = 25 μm. **(C)** Quantification of β-tubulin and Iba1 protein in the optic nerve head (ONH) and isolated ONH astrocytes using capillary electrophoresis. Neither β-tubulin nor Iba1 was detected in the ONH astrocyte cultures (square symbols); *n* = 3 biological replicates per group. **(D)** Quantitative PCR for a number of glial transcripts and transcripts for retinal ganglion cells (*Rbpms*), microglia (*Iba1*), and oligodendrocytes (*Mbp*) was used to further characterize the ONH astrocytes. *Rbpms*, *Iba1*, and *Mbp* were not detected in mRNA isolated from control or stretched ONH astrocytes (ND). There were no differences in transcripts for *Gfap*, *Slc1a3* (glutamate-aspartate transporter), *Glud1* (glutamate dehydrogenase-1), and *Glul* (glutamine synthetase). There were significant differences in the *Gja1* transcript levels, with stretched ONH astrocytes showing greater fold change as compared to control (*p = 0.0152, *n* = 3). *Gja1* encodes Connexin-43; *n* = 3 biological replicates per group. **(E)** GFAP protein, increased when astrocytes undergo hypertrophic reactivity, did not statistically differ between control and stretched ONH astrocytes; *n* = 3 biological replicates per group. **(F)** S100β protein, also upregulated in reactive astrocytes, did not statistically differ between control and stretched ONH astrocytes; *n* = 3 biological replicates per group.

**FIGURE 2:**
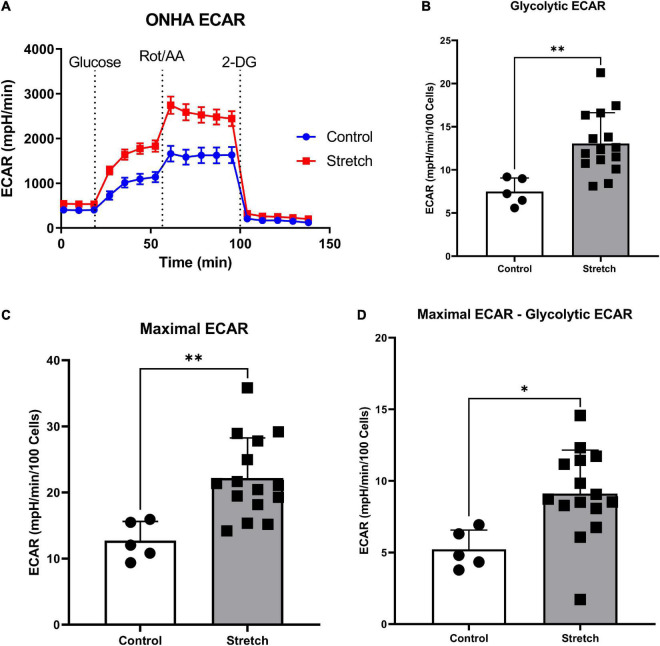
*Glaucoma-like stretch causes an increase in glycolytic activity in primary optic nerve head astrocytes (ONHAs).* **(A)** Stretch increases the extracellular acidification rate (ECAR) of primary ONH astrocytes. Measurements were taken every 9 min for baseline OCR, then addition of glucose, rotenone with Antimycin-A, and 2-deoxyglucose (2-DG). **(B)** Following the addition of glucose, stretched ONH astrocytes have a significantly higher ECAR than control ONH astrocytes (**p = 0.0037, *n* = 20). **(C)** Stretched ONH astrocytes have a significantly higher maximal ECAR than control ONH astrocytes (**p = 0.0039, *n* = 20). **(D)** Stretched ONH astrocytes have a significantly higher ECAR upregulation following the addition of rotenone and Antimycin-A compared to control ONH astrocytes (*p = 0.0136, *n* = 20). Values for panels **(B–D)** are normalized to cell number.

**FIGURE 3:**
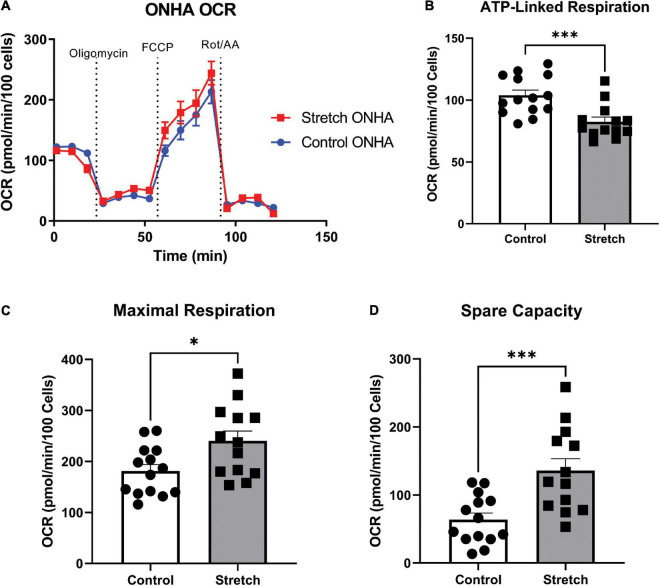
*Optic Nerve Head Astrocytes (ONHAs) stretched as in glaucoma have altered mitochondrial respiration compared to control ONHAs*. **(A)** Stretched ONH astrocytes have no difference in basal oxygen consumption rate (OCR) when compared to Control. Graph shows one representative example of OCR taken from the three biological replicates analyzed over 20–30 wells. **(B)** Stretched ONH astrocytes have a significantly lower ATP-linked mitochondrial OCR than control ONH astrocytes (***p = 0.0009, *n* = 27). **(C,D)** Stretched ONH astrocytes have a higher maximal respiration and spare capacity than control ONH astrocytes (*p = 0.0157, *n* = 27; ***p = 0.0010, *n* = 27).

**FIGURE 4:**
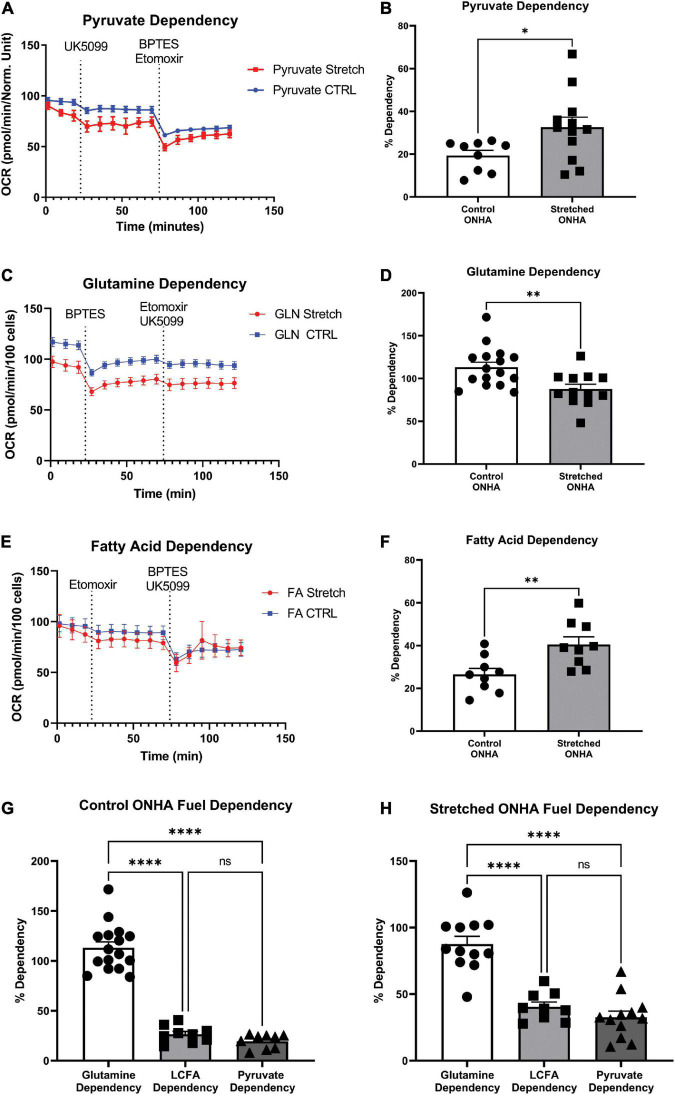
*Mitochondrial substrate dependency of optic nerve head astrocytes (ONHAs) is changed by glaucomic stretch*. **(A)** Representative oxygen consumption rate (OCR) output for the pyruvate dependency test. Cells are exposed to the mitochondrial pyruvate carrier inhibitor UK5099 followed by the glutamine and fatty acid oxidation pathway inhibitors BPTES and Etomoxir, respectively. **(B)** OCR for pyruvate dependency is significantly higher for stretched ONH astrocytes compared to Control (*p = 0.0347, *n* = 20). **(C)** Representative oxygen consumption rate (OCR) output for the glutamine dependency test. Cells are exposed to the glutaminase inhibitor BPTES followed by the fatty acid oxidation and pyruvate pathway inhibitors Etomoxir and UK5099, respectively. **(D)** Glutamine dependency is significantly lower in Stretched ONH astrocytes compared to Control (**p = 0.0052, *n* = 27). **(E)** Representative OCR output for the fatty acid dependency test. Cells are exposed to the carnitine palmitoyl-transferase 1a inhibitor Etomoxir followed by the glutamine and pyruvate pathway inhibitors BPTES and UK5099, respectively. **(F)** Fatty acid metabolic dependency is significantly higher in Stretched compared to Control ONH astrocytes (**p = 0.0071, *n* = 17). **(G)** Control ONHA fuel dependency is significantly different by one-way ANOVA (*p* < 0.0001). Control ONH astrocytes are significantly more dependent on glutamine as compared to fatty acids (****p < 0.0001) or pyruvate (****p < 0.0001) for fuel. These control ONH astrocyte data are the same as in panels **(B–F)**, collected together for ease of comparison. **(H)** Stretch ONHA fuel dependency is significantly different by one-way ANOVA (*p* < 0.0001). Stretched ONH astrocytes are also significantly more dependent on glutamine as compared to fatty acids (****p < 0.0001) or pyruvate (****p < 0.0001) for fuel. These stretch ONH astrocyte data are the same as in panels **(B–F)**, collected together for ease of comparison.

## Results

### Optic nerve head astrocyte characterization

Optic nerve head astrocytes from the initial cell isolations were grown on coverslips to facilitate characterization by immunocytochemistry, protein analysis by capillary electrophoresis, and by quantitative PCR. Figure 1A shows the ONH astrocytes fully confluent and immunolabeled with astrocyte marker GFAP, neuronal marker β-tubulin, and microglial marker Iba1. No cells were observed to express either β-tubulin or Iba1. Tissue from mouse optic nerve head and optic nerve were also immunolabeled with GFAP, β-tubulin, and Iba1 for use as a positive control (Figure 1B). Quantification of β-tubulin and Iba1 in the optic nerve head (positive control) and ONH astrocytes also showed no measurable β-tubulin or Iba1 in the ONH astrocytes (ONHA), Figure 1C. We used isolated mRNA from the control and stretched ONH astrocytes to characterize culture purity, finding that transcripts such as *Rbpms* (a retinal ganglion cell gene), *Iba1* (specifically found in microglia), and *Mbp* (myelin basic protein found in oligodendrocytes) were not detected in the control and stretched ONH astrocyte cultures (Figure 1D). There were no statistically significant differences in transcripts between the control and stretch ONH astrocytes with the exception of *Gja1*, the gene that encodes Connexin-43. Stretched ONH astrocytes had significantly higher *Gja1* transcript levels as compared to control (*p* = 0.0152, *n* = 3).

Biaxial stretch (12% stretch for 24 h, see Methods for more detail) of the ONH astrocytes resulted in increased GFAP and S100β protein in the stretched ONH astrocytes, though these changes were not statistically significant (Figures 1E,F), likely a reflection of the relatively short duration of stretch injury. Both GFAP and S100β are upregulated in reactive astrocytes (Cerutti and Chadi, 2000; Sofroniew, 2005).

### Glycolytic rate assay

We first exposed stretched and control ONH astrocytes to a glycolytic stress test in order to compare the glycolytic rate, as measured by extracellular acidification rate (ECAR) following exposure to electron transport chain (ETC) inhibitors rotenone (Complex I) and antimycin-A (Complex III). ECAR data normalized to cell number showed no difference in baseline ECAR between stretched and control ONH astrocytes (Figure 2A; *p* = 0.770, *n* = 20). Due to the absence of glucose in the media, this extracellular acidification comes from cell processes other than glycolysis, mainly oxidative respiration (Wu et al., 2007; Mookerjee et al., 2015). Once glucose was added, stretched ONH astrocytes had higher glycolytic ECAR values compared to control (Figure 2B, *p* = 0.0037, *n* = 20), suggesting that the stretched ONH astrocytes are more glycolytically active than control astrocytes. Maximal ECAR, the degree to which the cells can increase their glycolytic activity to produce ATP when mitochondrial respiration is inhibited, was significantly higher in stretched compared to control ONH astrocytes (Figure 2C, *p* = 0.0039, *n* = 20). Stretched ONH astrocytes also had a higher glycolytic reserve than control ONH astrocytes, as determined by the difference between maximal and glycolytic ECAR (Figure 2D, *p* = 0.0136, *n* = 20). These data indicate the stress caused by biaxial stretch of the ONH astrocytes expanded energy utilizing processes.

### Mitochondrial stress test

To determine to what degree the stretched ONH astrocytes utilized their mitochondria, we exposed ONH astrocytes to the mitochondrial stress test, and assay that uses electron transport chain complex inhibitors to challenge mitochondrial respiration as measured by oxygen consumption rate (OCR) in the Seahorse Analyzer. Stretched ONH astrocytes had no difference in their basal respiration compared to control ONH astrocytes (Figure 3A). Inhibition of the ATP synthase using oligomycin-A showed that stretched ONH astrocytes had lower ATP-linked respiration compared to control ONH astrocytes (Figure 3B, *p* = 0.0009, *n* = 27). Despite this difference in ATP-linked respiration, stretched ONH astrocytes had higher maximal respiration (Figure 3C, *p* = 0.0157, *n* = 27) and higher spare capacity (Figure 3D, *p* = 0.0010, *n* = 27), than control ONH astrocytes. This suggests that the stretched ONH astrocytes have the capacity to be more responsive to metabolic challenges than control ONH astrocytes.

### Mitochondrial fuel dependency test

Cells under stress may be inclined to shift their ability to use energy substrates for fuel, including expanding their capacity to use certain substrates. To test this hypothesis, we used the mitochondrial fuel dependency test on three possible fuel sources, pyruvate, glutamine, and long chain fatty acids. Compounds that can block transport or conversion of each of these fuel sources are used to observe how OCR changes under their influence. A large decrease in OCR after application of UK5099, an inhibitor of the mitochondrial pyruvate carrier, for example, suggests pyruvate as an important source of mitochondrial fuel for ONH astrocytes. Inhibitors of glutaminase for glutamine conversion to glutamate (BPTES), and carnitine palmitoyl-transferase 1A for long chain fatty acid translocation into the mitochondria for β-oxidation (Etomoxir) were used to evaluate dependence on glutamine and long chain fatty acids, respectively (Figures 4A–C). Representative OCR plots for pyruvate, glutamine, and long chain fatty acids are shown (Figures 4A–C). Stretched ONH astrocytes significantly increased their dependence on pyruvate compared to control (*p* = 0.0347, *n* = 12), Figure 4B. Dependence on glutamine decreased significantly in the stretched ONH astrocytes compared to control (*p* = 0.0052, *n* = 16), Figure 4D. Stretched ONH astrocytes significantly increased their reliance on fatty acids, as shown in Figures 4E,F (*p* = 0.0071, *n* = 9).

Interestingly, the stretched ONH astrocytes increased their dependency on pyruvate and fatty acids compared to control ONH astrocytes, but the relative dependence of specific substrates within groups remained equivalent. For example, both control and stretched ONH astrocytes were dependent upon glutamine to degree that was significantly greater than either long chain fatty acid or pyruvate (Figures 4G,H; *p* < 0.0001 for glutamine comparisons to either long chain fatty acid or pyruvate). There was no difference in dependence on long chain fatty acid or pyruvate within the control or stretched ONHA groups. These data indicate that dependency on pyruvate and fatty acids expanded, making up the share of fuel that was not coming from glutamine in stretched ONH astrocytes.

### Optic nerve head astrocyte protein analysis

**FIGURE 5:**
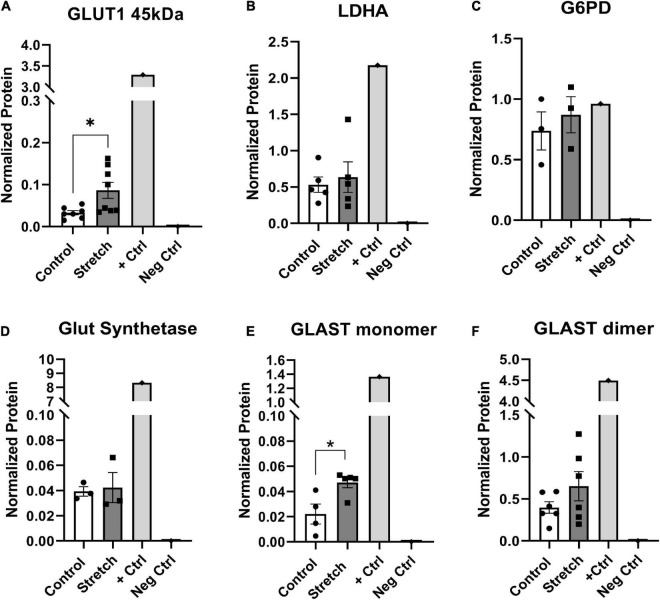
*Protein changes in ONHAs corroborate bioenergetics data*. **(A)** Glucose transporter-1 protein levels in Stretched ONH astrocytes are significantly higher than Control (*p = 0.0225, *n* = 7 Control, *n* = 8 Stretch). Retinal lysate from a 2 month-old mouse was used as a positive control for each protein analyzed, while negative control was the signal obtained when no primary antibody was included in the capillary. **(B)** Lactate dehydrogenase-A, the astrocyte-specific isoform of the enzyme that catalyzes the interconversion of pyruvate and lactate, has equivalent protein levels in Control and Stretch ONH astrocytes. **(C)** Glucose-6-phosphate dehydrogenase, the enzyme that shunts glucose into the pentose phosphate pathway, is no different in Control and Stretch ONH astrocytes. **(D)** Glutamine synthetase, the enzyme that synthesizes glutamine from glutamate, is no different in Control and Stretch ONH astrocytes. **(E)** The monomeric form of glutamate-aspartate transporter (GLAST) has significantly higher protein levels in the Stretch as compared to the Control ONH astrocytes (*p* = 0.020; *n* = 4 Control, *n* = 5 Stretch). **(F)** GLAST dimer protein levels are no different in Control and Stretch ONH astrocytes.

The metabolic alterations we observed in ONHA function after stretch suggest we might also identify corresponding changes in metabolism-associated proteins in these cells. For example, glutamine dependency might suggest potential changes in glutamate-aspartate transporter (GLAST), or glutamine synthetase. There was a significant increase in the protein levels of glucose transporter-1 (GLUT1) in the stretched ONH astrocytes as compared to control; the difference was noted in the astrocyte-specific 45kDa isoform of the transporter (*p* = 0.0225, Figure 5A). This suggests that stretched astrocytes had an increased capacity for the uptake of glucose. There was no difference in protein levels for the astrocyte-specific lactate dehydrogenase (Figure 5B); glucose-6-phosphate-dehydrogenase (Figure 5C), the enzyme that shunts glucose into the pentose-phosphate pathway; or glutamine synthetase (Figure 5D), the enzyme that converts glutamate to glutamine. However, the glutamate-aspartate transporter (GLAST) protein in monomeric form was significantly increased in the Stretch group (*p* = 0.020). GLAST dimers were prominent in the analyzed proteins, but did not differ across the Control and Stretch groups.

## Discussion

Isolating ONH astrocytes, subjecting them to biaxial stretch then evaluating their bioenergetics using the Seahorse Analyzer has yielded insight into astrocytic metabolic changes as a result of mechanical stress. To the degree that these mechanical stresses recapitulate the strain experienced by the cells of the ONH during ocular hypertension, we have gained insight into the metabolic shifts and fuel preferences of ONH astrocytes in glaucoma.

Optic nerve head astrocytes subjected to stretch increase their extracellular acidification when glucose is made available, but also after electron transport chain (ETC) disruption. This likely reflects an overall increase in metabolic demand as a result of mechanical stress. Interestingly, stretched ONH astrocytes had higher maximal respiration and spare capacity compared to control cells, but lower ATP-linked respiration. Finally, we showed that stretched ONH astrocytes were more dependent on pyruvate and fatty acids as fuel compared to control ONH astrocytes, but this did not significantly alter their significant reliance on glutamine. Both control and stretched ONH astrocytes exhibit a preference for glutamine.

Despite having extensive mitochondrial networks, astrocytes are primarily reliant on glycolysis (Rahman and Suk, 2020) for their ATP production than on mitochondrial respiration (Dienel and Hertz, 2001; Turner and Adamson, 2011). Consistent with this, extracellular acidification rate (ECAR) in the stretched and control ONH astrocytes was robust. The stretched ONH astrocytes exhibited significantly greater ECAR than control in the presence of glucose and after inhibition of Complexes I and III of the electron transport chain (ETC). Our interpretation of these data is that the stretched ONH astrocytes were much more metabolically active than control cells. Our ECAR data does not distinguish between proton release from lactate extrusion versus from the tricarboxylic acid cycle, though with glucose as the primary substrate, the contribution of ECAR from glycolysis often outpaces that from the TCA cycle (Mookerjee et al., 2015). Lamina cribrosa cells isolated from ONHs of patients with glaucoma showed increased lactate-associated extracellular acidification compared to control patient lamina cribrosa (LC) cells, suggesting increased glycolytic flux with glaucoma (Kamel et al., 2020). Our findings are consistent with the LC observations. Whereas the human LC cells are not astrocytes, the two cell types have overlapping roles in the ONH since rodents possess a glial lamina composed of astrocytes (Hernandez, 2000b; Sun et al., 2009).

The increased ECAR after glucose addition in the stretched ONH astrocytes might have been anticipated to accompany an increase in lactate dehydrogenase (LDH) since ECAR is primarily a result of lactate release, which co-transports a proton, thereby acidifying the extracellular milieu. In many contexts, astrocytes are a source of lactate to fuel/support the neurons in their vicinity (Pellerin et al., 1998; Volkenhoff et al., 2015; Muraleedharan et al., 2020). However, we observed no changes in the LDH protein when comparing control and stretched ONH astrocytes. One possible explanation is that the dual role of LDH, the interconversion of pyruvate and lactate, precludes a linear relationship between lactate production and LDH protein levels. We specifically probed for LDHA, the isoform that preferentially converts pyruvate into lactate (O’Brien et al., 2007). Another possibility is that LDH enzymatic activity might have been increased in stretched ONH astrocytes without a change in the overall protein. ECAR was not different between control and stretched ONH astrocytes prior to the addition of glucose, so LDH stability is consistent with the data indicating stretched ONH astrocytes are more dependent on glutamine than pyruvate. Proteomics undertaken on human ONH astrocytes subjected to 2h of 3% stretch showed a downregulation of lactate dehydrogenase A-like 6B protein (Rogers et al., 2012a), but this change was not observed when the degree of stretch and length of time at stretch were increased to values that matched our own experiment.

### Stretched optic nerve head astrocyte mitochondria are more responsive than control

Stretched ONH astrocytes showed increased maximal respiratory rate and spare capacity compared to control ONH astrocytes. We observed no difference in the baseline mitochondrial OCR in stretch and control ONHA groups. These observations differ from those made of glaucoma patient LC cells, which showed significantly lower basal OCR and spare capacity compared to control patient LC cells (Kamel et al., 2020). A key difference is that LC cells from glaucoma patients are likely to have had chronic exposure to strain, while our ONH astrocytes were under biaxial strain for 24 h only. In microarray analysis of human glaucomatous ONHA, genes associated with ATP metabolism were downregulated (Nikolskaya et al., 2009). Though not detailed enough to indicate which metabolic genes in particular were affected, the ATP metabolism gene downregulation could be consistent with the significantly lower ATP-linked respiration we observed in stretched ONH astrocytes compared to control. Since ECAR data indicates greater metabolic activity in the stretched cells, these observations indicate the stretched cells obtained slightly greater metabolic support from glycolysis than the control ONH astrocytes. This is corroborated by the increased pyruvate dependency demonstrated by the stretched ONH astrocytes. The stretched ONH astrocytes had greater maximal respiration and spare capacity than control, indicating that the biaxial strain has promoted a metabolic resilience or responsiveness in the cells such that they should be able to meet additional energy demand when called upon to do so. The increased dependency on fatty acids for energy by the stretched cells is consistent with studies showing that reactive astrocytes increase their fatty acid metabolism (Zamanian et al., 2012; Liddelow et al., 2017). The ability to utilize fatty acids contributes to the metabolic resilience. Fuel flexibility and resilience are positive, though *in vivo* would be limited by the eventual structural breakdown of ONH astrocytes that has been observed in rat (Dai et al., 2012) and mouse (Lye-Barthel et al., 2013) ONH in the course of glaucoma pathogenesis.

### Stretched optic nerve head astrocytes had altered mitochondrial fuel dependency

The dependency of ONH astrocytes for each of the three fuel sources we tested differed across stretched and control ONH astrocytes. Stretched ONH astrocytes had significantly greater dependency on pyruvate and fatty acids than controls ONH astrocytes. Dependence of stretched ONH astrocytes on glutamine decreased compared to control. However, since the dependence of both stretched and control ONH astrocytes was nevertheless overwhelmingly for glutamine, we interpret the shift toward pyruvate and fatty acid for the stretched ONH astrocytes as revealing an expansion of their possible means by which to meet their increased metabolic needs.

Optic nerve head astrocyte dependence on glutamine over fatty acids or pyruvate was somewhat unexpected given the emphasis on astrocyte glycolysis as opposed to respiration to meet metabolic needs (Dienel and Hertz, 2001; Turner and Adamson, 2011). Our data suggest the glutamate-glutamine cycle plays a much larger role in ONHA metabolism than previously appreciated. Astrocytes transport glutamate from the extracellular space using GLAST and GLT-1, a critical management of excitatory neurotransmitters (Robinson and Jackson, 2016). Glutamate is converted to glutamine through the activity of glutamine synthetase. We observed no difference in glutamine synthetase (GS) protein in control versus stretched ONH astrocytes. At first glance, this could suggest that imported glutamate may not be the source of glutamine for these ONH astrocytes; glutamine was available directly from the cell media. However, we observed significant upregulation of GLAST in the stretched ONH astrocytes. The capacity for glutamate import increased in stretched ONH astrocytes, and it may be the case that GS activity increased to address the greater concentrations of glutamate without altering overall GS protein. Alternatively, the dependency on glutamine may be masking a use of glutamate by the ONH astrocytes. Glutamate can be oxidized via glutamate dehydrogenase, or oxidized to α-ketoglutarate for the TCA cycle via aspartate aminotransferase (Daikhin and Yudkoff, 2000). In fact, glutamate could theoretically substitute for glucose for fuel. In human LC cells from glaucoma patients, there was a significant upregulation of glutaminase-2 (Kamel et al., 2020), an enzyme that converts glutamine to glutamate. We did not measure glutaminase-2, but anticipate that it may be increased in stretched ONH astrocytes. If ONH astrocytes are using glutamine to generate glutamate that supplies the TCA cycle with carbon, then we would anticipate that these cells would not be exporting glutamine for uptake by neurons. This is testable by measuring amino acid transporter Slc38a1 protein or activity (Bröer and Brookes, 2001). Interestingly, fatty acids are critical for the success of the glutamate-glutamine cycle because the amino groups provide the nitrogen for the production of glutamine from glutamate (Daikhin and Yudkoff, 2000). This may explain the increased metabolic dependency of stretched ONH astrocytes on fatty acids we observe.

Might the ONH astrocytes generate glutamine only to convert it to glutamate for fuel when it is not taken up by neurons? At high external concentrations of glutamate, the entry of glutamate into the TCA cycle in astrocytes is favored over conversion to glutamine (McKenna et al., 1996). What is unclear for our experiments is the source of glutamate since these cultures did not include cells other than the ONH astrocytes. Relatedly, the dependence of ONH astrocytes in general on glutamine in our experiments raises the issue of whether their metabolic choices are governed by being a monoculture. It would not be surprising if ONH astrocytes do not prefer glucose for fuel as a result of not having neurons to which they can easily pass lactate. Future experiments that co-culture ONH astrocytes with neurons could resolve this question. The upregulation in GLAST and the distinct reliance of ONH astrocytes on glutamine metabolism also indicates a larger role for mitochondrial respiration in the stretched ONH astrocytes. The astrocytic preference for glutamine over pyruvate as a mitochondrial fuel source reinforces the lactate-shuttle hypothesis (Pellerin et al., 1998) since not relying on pyruvate for mitochondrial respiration allows them to export lactate.

In our protein analysis to corroborate our bioenergetic findings, we observed no increase in glucose-6-phosphate-dehydrogenase, the enzyme that shunts glucose into the pentose phosphate pathway. This suggests that there is no change in the management of metabolites into the pentose phosphate pathway, which may also be consistent with the emphasis of these ONH astrocytes on utilizing glutamine for fuel. The absence of this shunting could ultimately be deleterious for the stretched ONH astrocytes since NADPH from the pentose phosphate pathway is used for the production of glutathione (Grant, 2008), an antioxidant effective at limiting reactive oxygen species (ROS). ROS production is upregulated in reactive astrocytes (Sheng et al., 2013; Vicente-Gutiérrez et al., 2021), so one might expect increased glutathione from the pentose phosphate pathway could assist in ROS management. In future experiments, we will evaluate antioxidant production in stretched ONH astrocytes.

We observed a significant increase in GLUT1 protein, the primary means by which astrocytes take up glucose from the vasculature and the parenchyma. In past studies, we have reported a significant decline in GLUT1 protein in the optic nerve (ON) of glaucomatous mice (Harun-or-Rashid et al., 2018). Timing is a consideration here, as we cannot easily compare ONH astrocytes exposed to 24 h of stretch to ONs from mice with 6 + months of increased IOP. Perhaps the difference between GLUT1 protein in the ON and ONH is one that requires a metabolic perspective and not just a structural one. GLUT1 upregulation serves the stretched ONH astrocytes well by importing more glucose. Our data indicates the stretched ONH astrocytes become more dependent on pyruvate, a situation that would be enabled by increased glucose transport. We have little reason to think that ON and ONH astrocytes are homogeneous. The glia lamina of the ONH is a unique structure with astrocytes oriented differently than those in the ON (Sun et al., 2009). While it has been shown that ONH astrocytes are connected by gap junctions (Quigley, 1977), it remains to be determined if the two populations of ON and ONH astrocytes are connected by gap junctions. *In vitro* experiments have shown that both hydrostatic pressure and oxygen-glucose deprivation and reperfusion led to decreases and relocation of connexin-43 intracellularly (Malone et al., 2007; Xie et al., 2017), potentially reducing the availability of glucose among astrocytes. We did not evaluate astrocyte coupling, but recognize the need to determine whether ON and ONH astrocytes form a network. The alternate roles and morphologies across the ON and ONH argue for heterogeneous populations of astrocytes. Increased GLUT1 protein in the stretched ONH astrocytes enables greater metabolic activity, likely to support the cellular alterations in cytoskeleton and function prompted by the strain experienced by the cells.

Stretched ONH astrocytes demonstrate metabolic changes that indicate the expansion of metabolic substrate utilization to support the increased metabolic activity noted in these cells. Future work will investigate the implications of these ONHA changes on retinal ganglion cells axon function.

## Data availability statement

The original contributions presented in this study are included in the article/Supplementary Material, further inquiries can be directed to the corresponding author.

## Ethics statement

The animal study was reviewed and approved by The Institutional Animal Care and Use Committee of UNTHSC.

## Author contributions

NP designed and performed experiments, analyzed data, created figures, and wrote the initial draft of the manuscript. EY, ABM, and CCK performed experiments and analyzed data. DMI designed experiments, analyzed data, created figures, edited the manuscript, and funded the research. All authors contributed to the article and approved the submitted version.
