# Supplementary material for: Stretch stress propels glutamine dependency and glycolysis in optic nerve head astrocytes
Source: Front Neurosci. 2022 Aug 5;16:957034. doi: 10.3389/fnins.2022.957034 (PMC9389405; doi:10.3389/fnins.2022.957034)
Supplement: Supplementary file 1 [file Data_Sheet_1.PDF]

Figure 1C

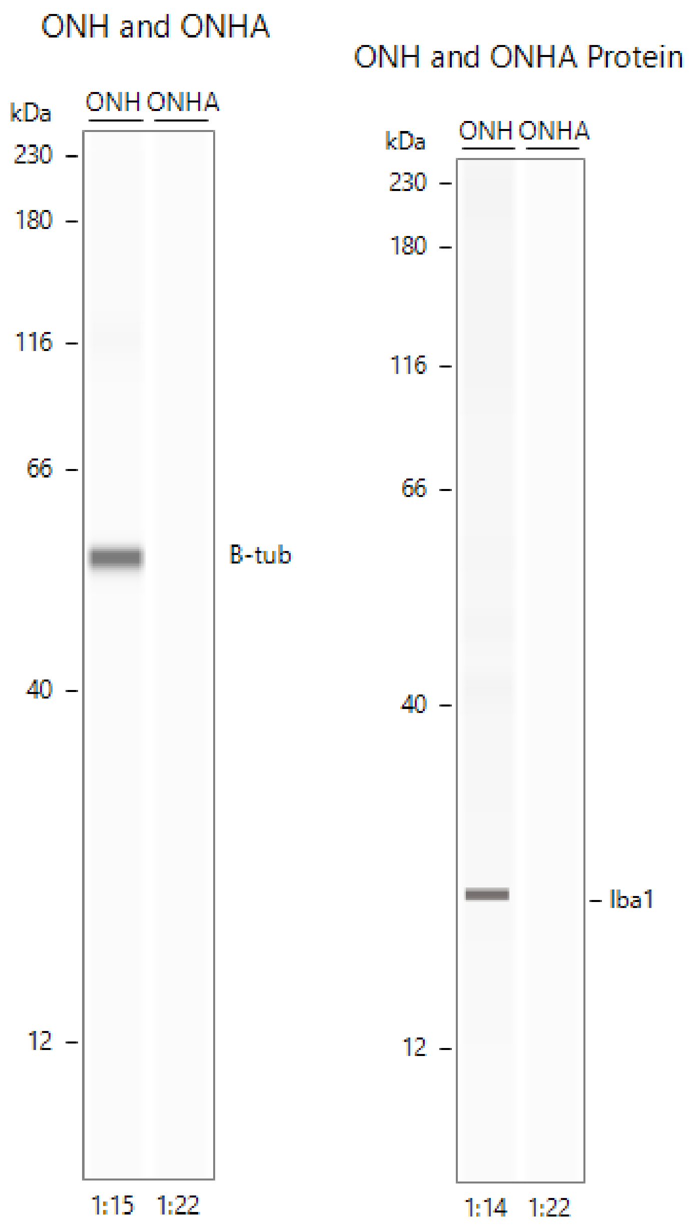
ONH and ONHA
kDa
ONH
ONHA
230
180
116
66
40
12
B-tub
1:15
1:22
ONH and ONHA Protein
kDa
ONH
ONHA
230
180
116
66
40
12
Iba1
1:14
1:22

Figure 1E and F

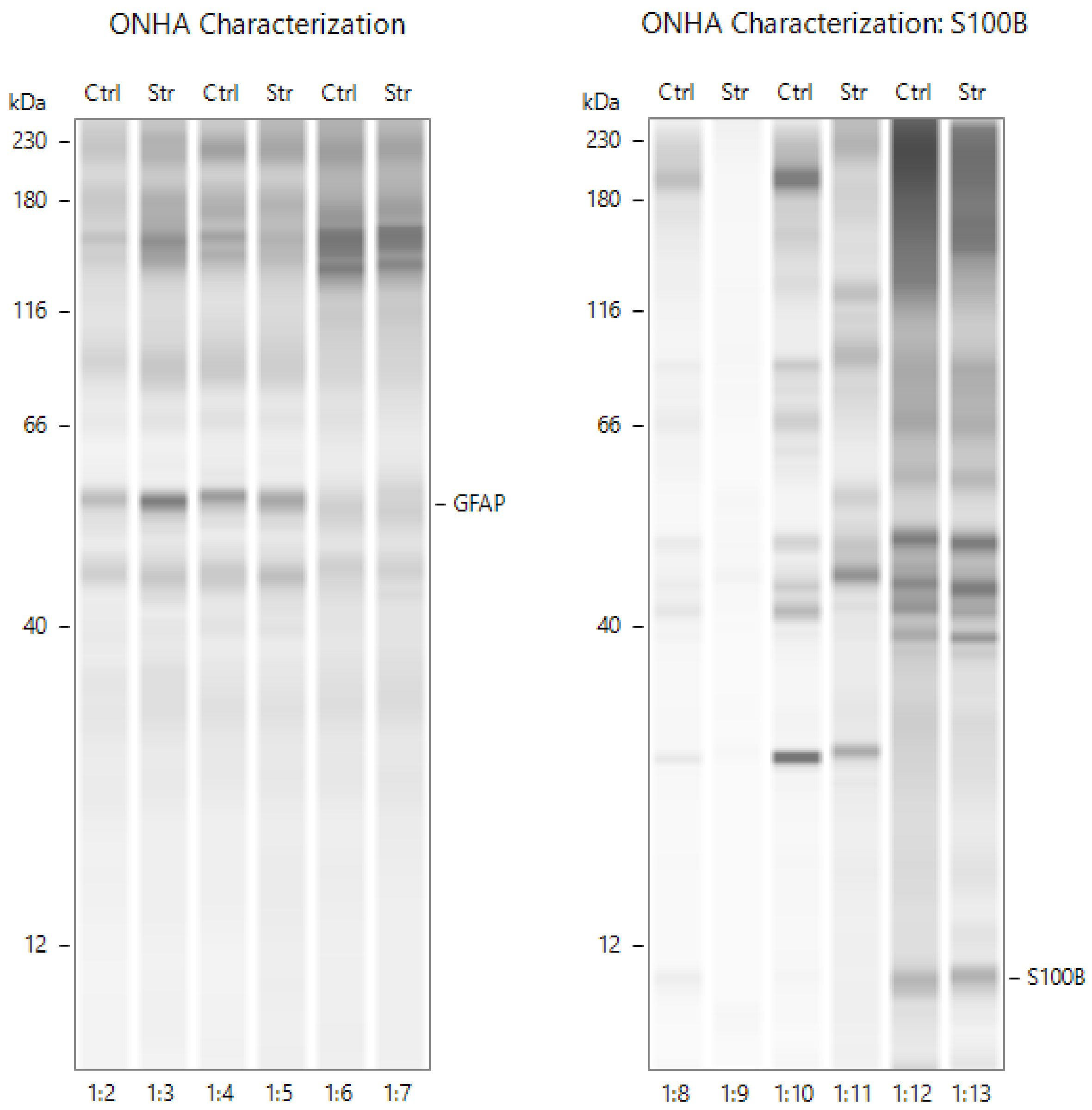

Figure 5A

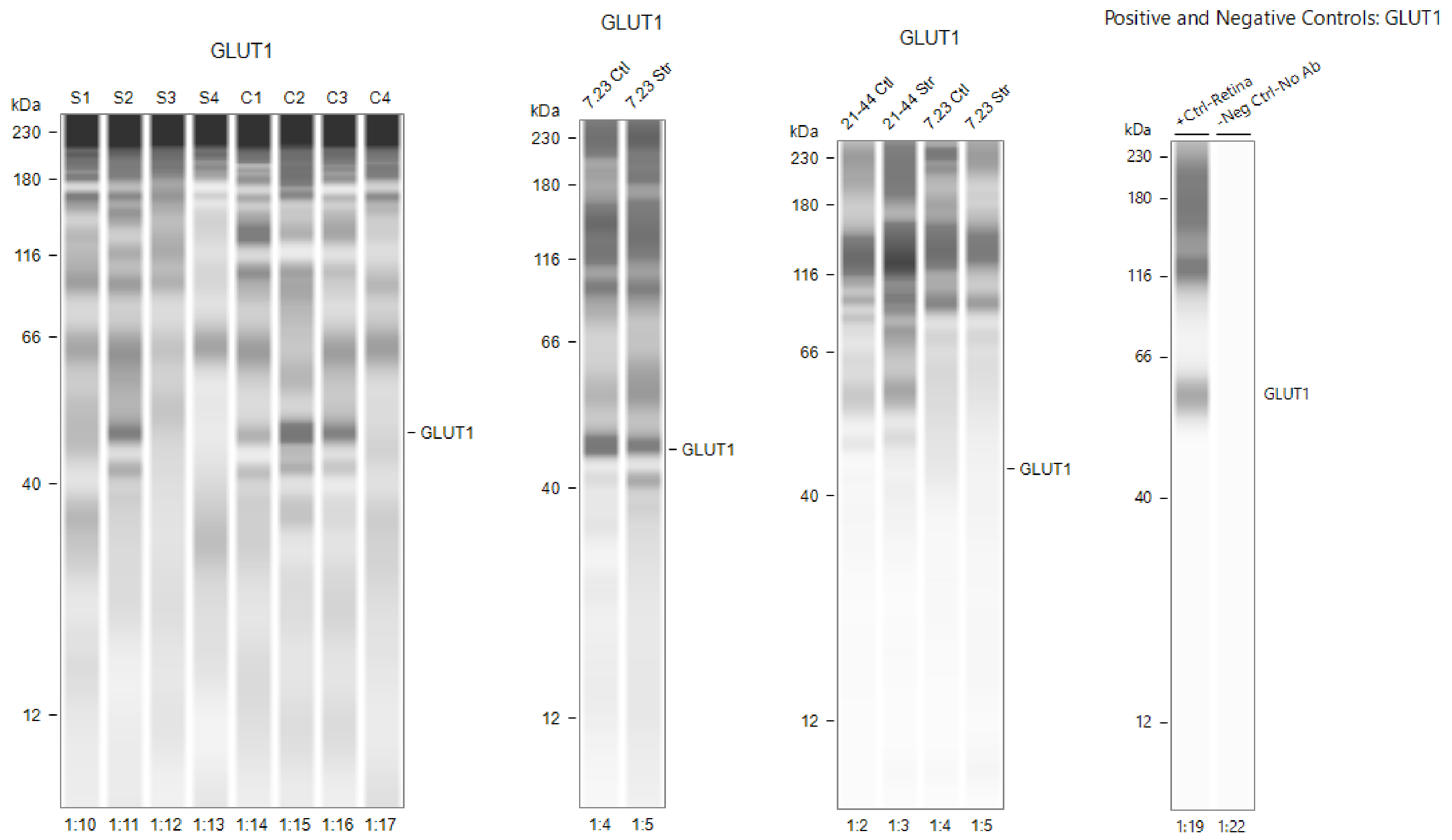
GLUT1
kDa
S1 S2 S3 S4 C1 C2 C3 C4
230
180
116
66
40
12
GLUT1
1:10 1:11 1:12 1:13 1:14 1:15 1:16 1:17
GLUT1
7.23 Ctl
7.23 Str
kDa
230
180
116
66
40
12
GLUT1
1:4 1:5
GLUT1
21-44 Ctl
21-44 Str
7.23 Ctl
7.23 Str
kDa
230
180
116
66
40
12
GLUT1
1:2 1:3 1:4 1:5
Positive and Negative Controls: GLUT1
+Ctrl-Retina
-Neg Ctrl-No Ab
kDa
230
180
116
66
40
12
GLUT1
1:19 1:22

Figure 5B

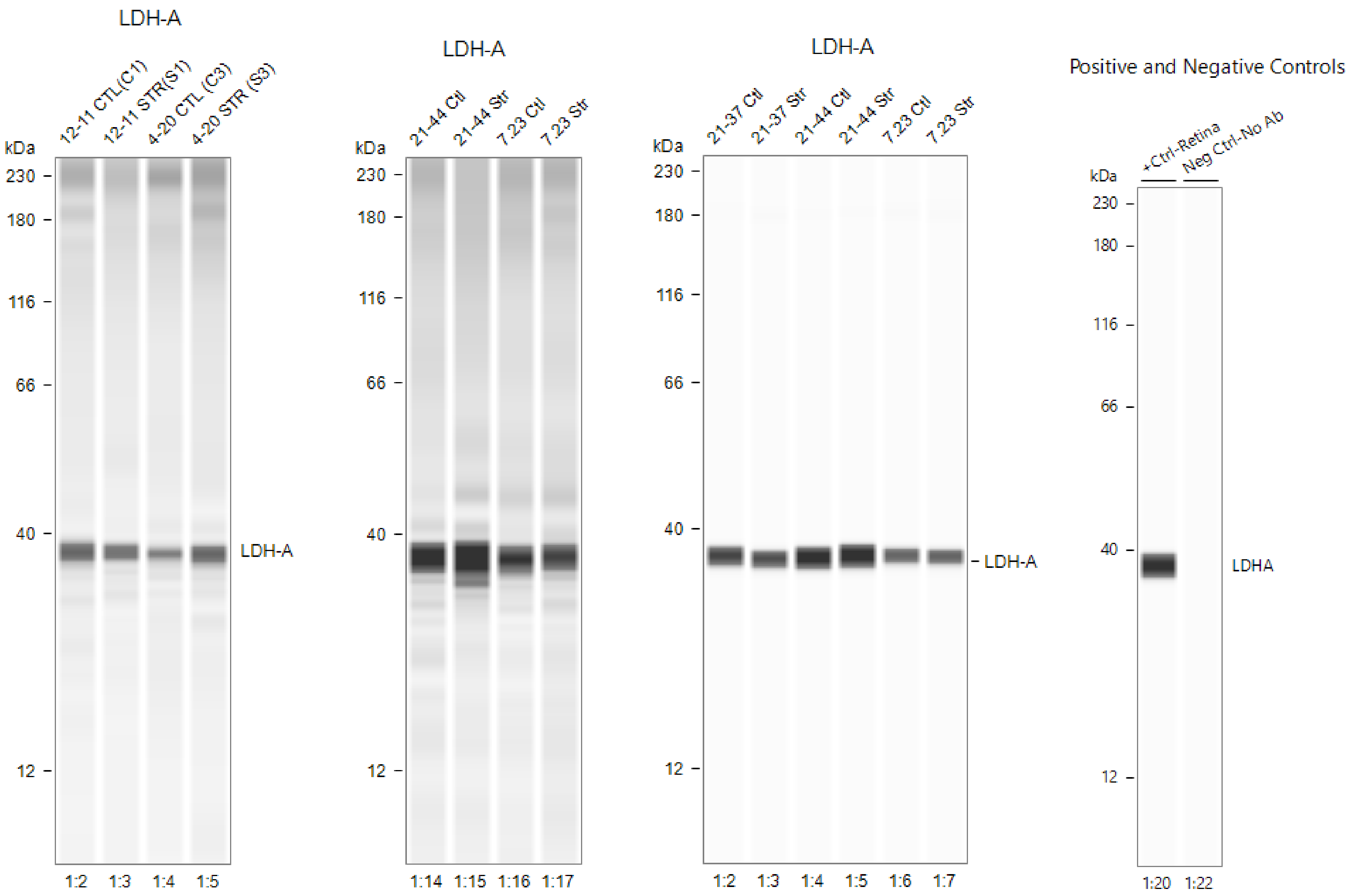
LDH-A
12-11 CTL(C1)
12-11 STR(S1)
4-20 CTL (C3)
4-20 STR (S3)
kDa
230
180
116
66
40
12
LDH-A
1:2 1:3 1:4 1:5
LDH-A
21-44 Ctl
21-44 Str
7.23 Ctl
7.23 Str
kDa
230
180
116
66
40
12
1:14 1:15 1:16 1:17
LDH-A
21-37 Ctl
21-37 Str
21-44 Ctl
21-44 Str
7.23 Ctl
7.23 Str
kDa
230
180
116
66
40
12
LDH-A
1:2 1:3 1:4 1:5 1:6 1:7
Positive and Negative Controls
+Ctrl-Retina
Neg Ctrl-No Ab
kDa
230
180
116
66
40
12
LDHA
1:20 1:22

Figure 5C

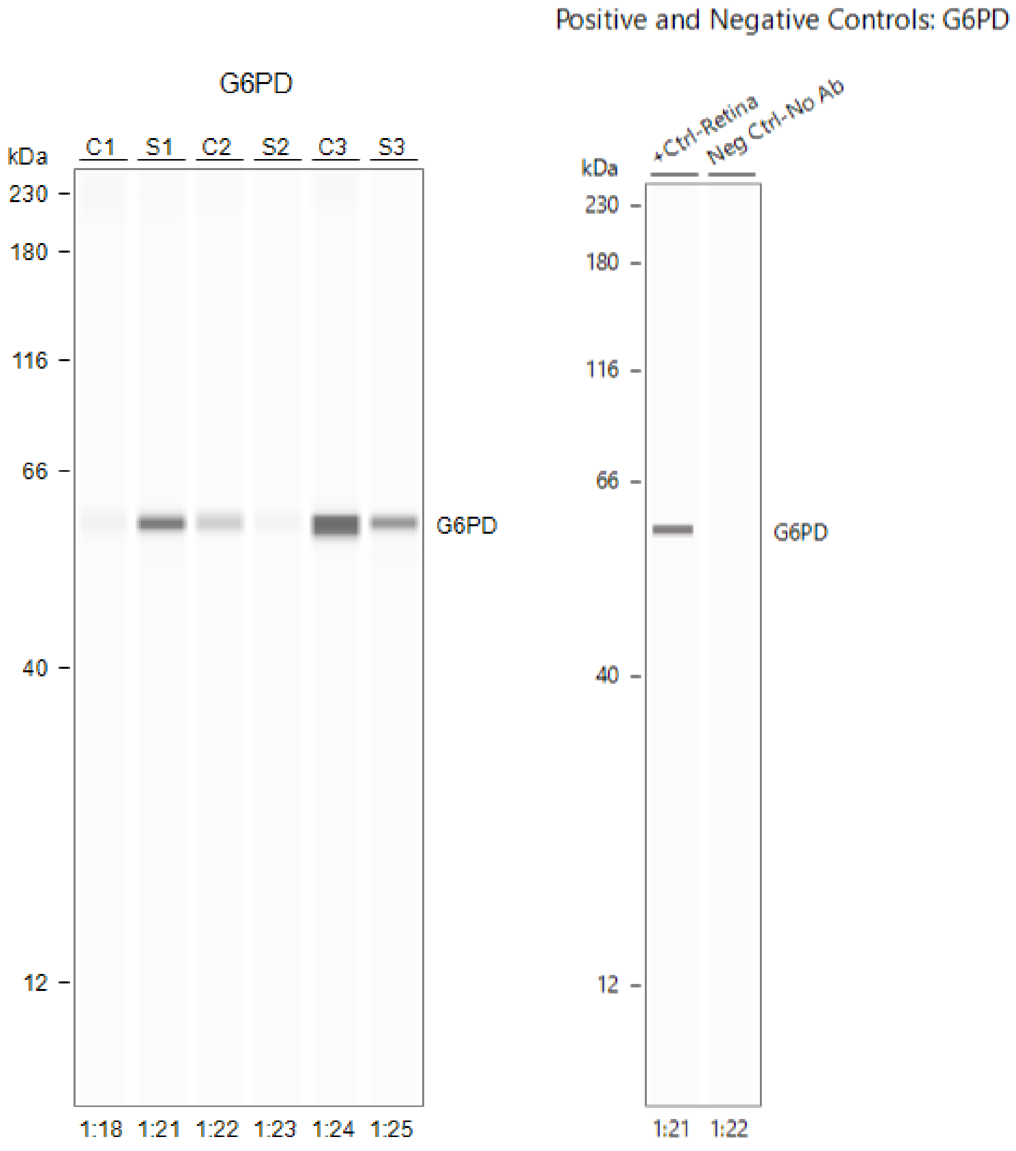
Positive and Negative Controls: G6PD
G6PD
kDa
C1
S1
C2
S2
C3
S3
230
180
116
66
40
12
G6PD
1:18
1:21
1:22
1:23
1:24
1:25
+Ctrl-Retina
Neg Ctrl-No Ab
kDa
230
180
116
66
40
12
G6PD
1:21
1:22

Figure 5D

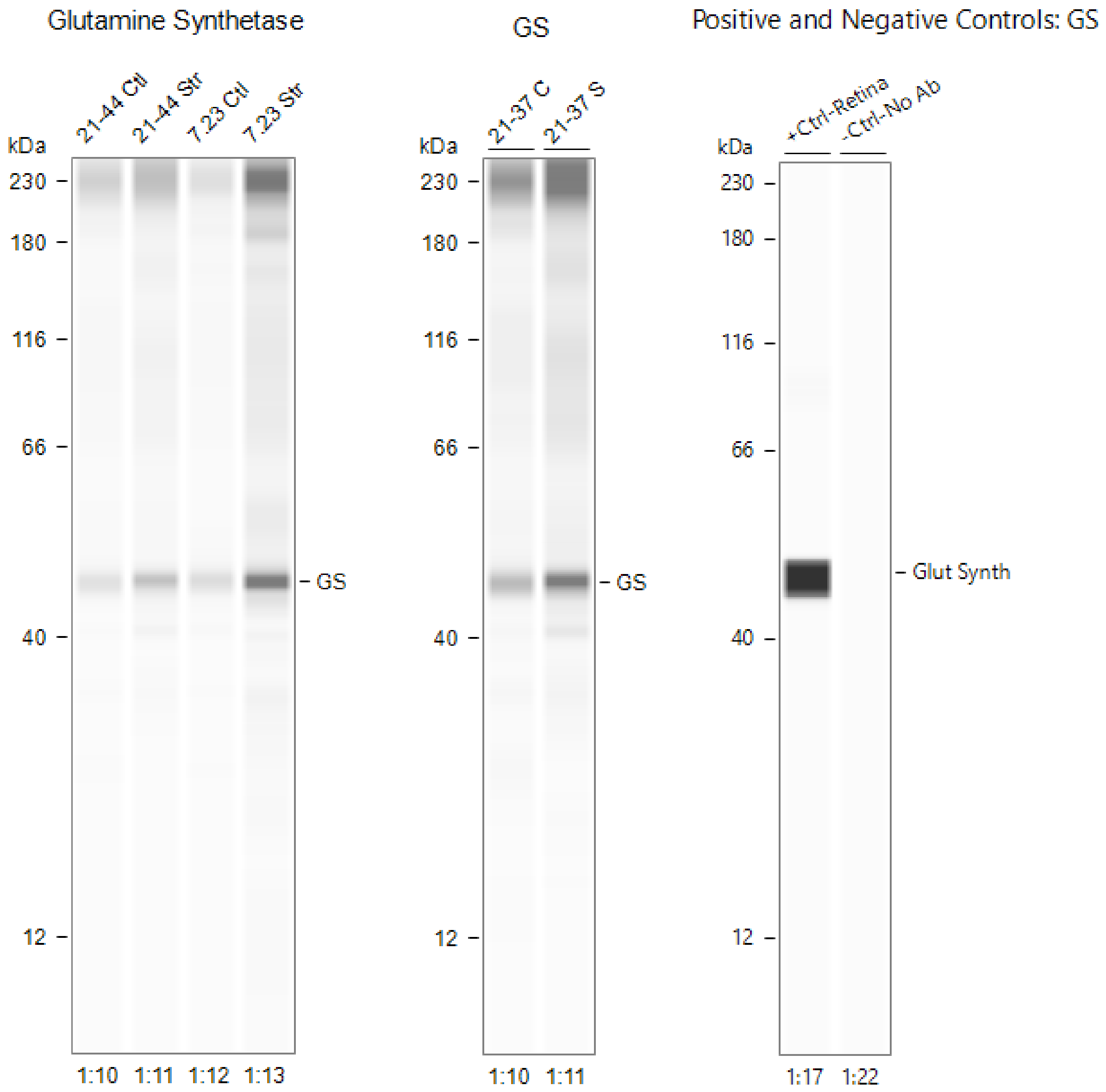

Glutamine Synthetase
21-44 Ctl
21-44 Str
7.23 Ctl
7.23 Str
kDa
230
180
116
66
40
12
GS
1:10
1:11
1:12
1:13
GS
21-37 C
21-37 S
kDa
230
180
116
66
40
12
GS
1:10
1:11
Positive and Negative Controls: GS
+Ctrl-Retina
-Ctrl-No Ab
kDa
230
180
116
66
40
12
Glut Synth
1:17
1:22

Figure 5E and F

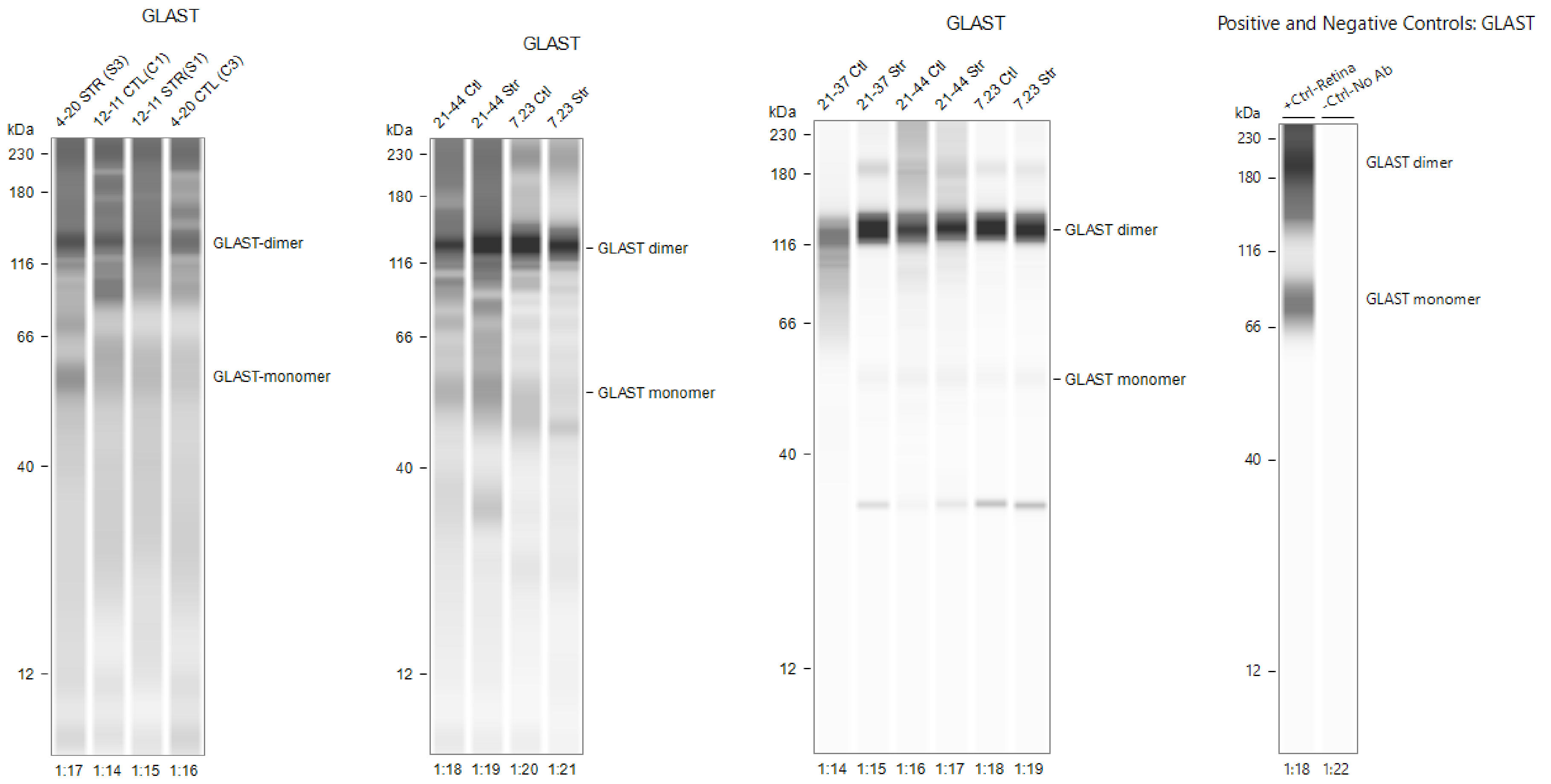

Note: GLAST monomer is visible when we increase the exposure time; these blots have been optimized to visualize the GLAST dimer.
